# Circadian Behaviour in Neuroglobin Deficient Mice

**DOI:** 10.1371/journal.pone.0034462

**Published:** 2012-04-05

**Authors:** Christian A. Hundahl, Jan Fahrenkrug, Anders Hay-Schmidt, Birgitte Georg, Birgitte Faltoft, Jens Hannibal

**Affiliations:** 1 Department of Clinical Biochemistry, Faculty of Health Science, Bispebjerg Hospital, University of Copenhagen, Copenhagen, Denmark; 2 Department of Neuroscience and Pharmacology, The Panum Institute, University of Copenhagen, Copenhagen, Denmark; Vanderbilt University, United States of America

## Abstract

Neuroglobin (Ngb), a neuron-specific oxygen-binding globin with an unknown function, has been proposed to play a key role in neuronal survival. We have previously shown Ngb to be highly expressed in the rat suprachiasmatic nucleus (SCN). The present study addresses the effect of Ngb deficiency on circadian behavior. Ngb-deficient and wild-type (wt) mice were placed in running wheels and their activity rhythms, endogenous period and response to light stimuli were investigated. The effect of Ngb deficiency on the expression of *Period1* (*Per1*) and the immediate early gene *Fos* was determined after light stimulation at night and the neurochemical phenotype of Ngb expressing neurons in wt mice was characterized. Loss of Ngb function had no effect on overall circadian entrainment, but resulted in a significantly larger phase delay of circadian rhythm upon light stimulation at early night. A light-induced increase in *Per1*, but not *Fos*, gene expression was observed in Ngb-deficient mice. Ngb expressing neurons which co-stored Gastrin Releasing Peptide (GRP) and were innervated from the eye and the geniculo-hypothalamic tract expressed FOS after light stimulation. No PER1 expression was observed in Ngb-positive neurons. The present study demonstrates for the first time that the genetic elimination of Ngb does not affect core clock function but evokes an increased behavioural response to light concomitant with increased *Per1* gene expression in the SCN at early night.

## Introduction

The brain's biological clock, located in the suprachiasmatic nucleus (SCN), generates circadian rhythm of physiology and behaviour. The clock needs daily adjustment (entraining) to stay synchronized with the astronomical day of 24 h. Daylight is the primary cue for this process known as photoentrainment [1]. Photoentrainment is a fundamental element of the circadian timing system and is dependent upon a functional retina, the SCN itself, as well as output signalling from the SCN [2]. Rhythmicity within the SCN is governed by a molecular clockwork, which operates in a subpopulation of SCN neurons located mainly in the dorsomedial part or shell region of the SCN [3], [4]. Other neurons located mainly in the ventrolateral part or core region of the SCN receive input for entrainment [5]. Neurons of the core region differ phenotypically and play different roles in the entrainment process. Neurons containing vasoactive intestinal peptide (VIP) and the VPAC2 receptor have been shown to play an essential role in the maintenance of ongoing circadian rhythmicity by synchronizing SCN cells and by maintaining oscillations within individual neurons [6]–[9]. Neurons expressing gastrin releasing peptide (GRP) and its receptors seem to be primarily involved in light induced resetting of the clock [10]–[12]. Recently, we demonstrated that neuroglobin (Ngb), a 17 kDa monomeric globin bearing structural resemblance to hemoglobin and myoglobin [13], is expressed in neurons of the rat SCN and found it to be co-stored with GRP in neurons of the ventro-lateral SCN [14], [15]. Ngb is evolutionarily older than both hemoglobin and myoglobin and can, as hemoglobin and myoglobin, reversibly bind oxygen with an affinity roughly similar to myoglobin [13], [16], [17]. Due to the neuronal localization and oxygen binding properties, Ngb has been proposed to be a novel oxygen reservoir of highly metabolic neurons [13]. Involvement of Ngb in protection against neuronal death and in signal transduction has also been suggested (for review see [18]). Within the rat SCN, most of the Ngb positive neurons are innervated from the geniculo-hypothalamic tract (GHT) [15] and express FOS after light stimulation at night [15]. A limited number of Ngb expressing neurons co-express the clock gene *PER1*, suggesting that Ngb neurons receive input for entrainment of the clock rather than being “clock cells”.

In the present study we have generated Ngb deficient mice to elucidate a possible role of Ngb in SCN physiology. Using this mouse model we provide evidence that Ngb is involved in light induced resetting of the clock but it is not necessary for core clock function.

## Results

### Animals

**Figure 1:**
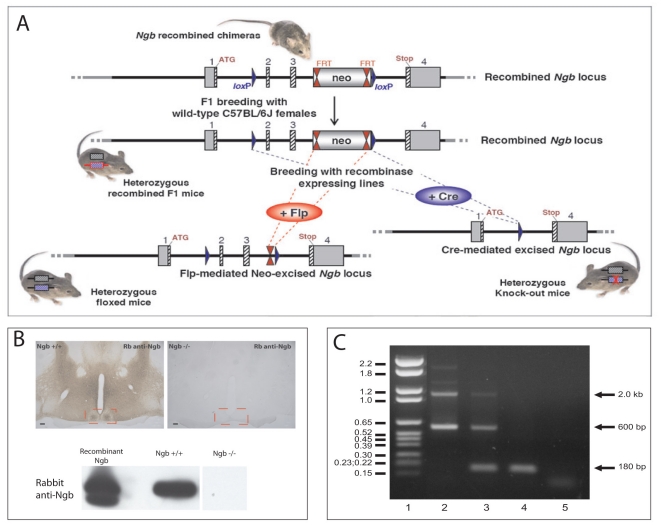
Generation and characterization of Ngb deficient mice. A. Schematic representation of the targeting construct introduced into 129Sv/Pas embryonic stem (ES) cells to generate Ngb-knockout mice. Chimeric mice were crossed with Flp recombinase-expressing mice to remove the Neo resistance cassette and obtain F1 founder mice. F1 founder mice were crossed with mice expressing Cre recombinase ubiquitously which resulted in the deletion of Ngb exons 2 and 3 (B–C). B. Coronal sections of Ngb^+/+^ and Ngb^−/−^ mice stained with rabbit anti-Ngb antiserum demonstrate specific staining in the Ngb wild type mouse and no staining in Ngb deficient mice. Similar results are obtained when tissue extracts from brains from the two genotypes were analyzed by western blotting (lower panel B). C represents PCR genotyping of littermates obtained by crossing heterozygous Ngb mice. Lane 1: DNA molecular marker VI (Roche), the sizes in kb are indicated into the left, lane 2: PCR on DNA from +/+ mouse, the expected size of bands are 2.0 kb and 600 bp, lane 3: PCR on DNA from +/− mouse, lane 4: PCR on DNA from Ngb −/− mouse, expected band size 180 bp; lane 5: Non-template control.

Ngb deficient animals were identified by PCR-based genotyping (Fig. 1C) and randomly tested by western blotting and immunohistochemistry. No Ngb staining was found in Ngb deficient mice using a well-characterized anti-Ngb antiserum (Fig. 1B). Ngb deficient mice exhibited no abnormalities in gross anatomy, body composition or overt behaviour.

### Behavioural studies of Ngb deficient mice

**Figure 2:**
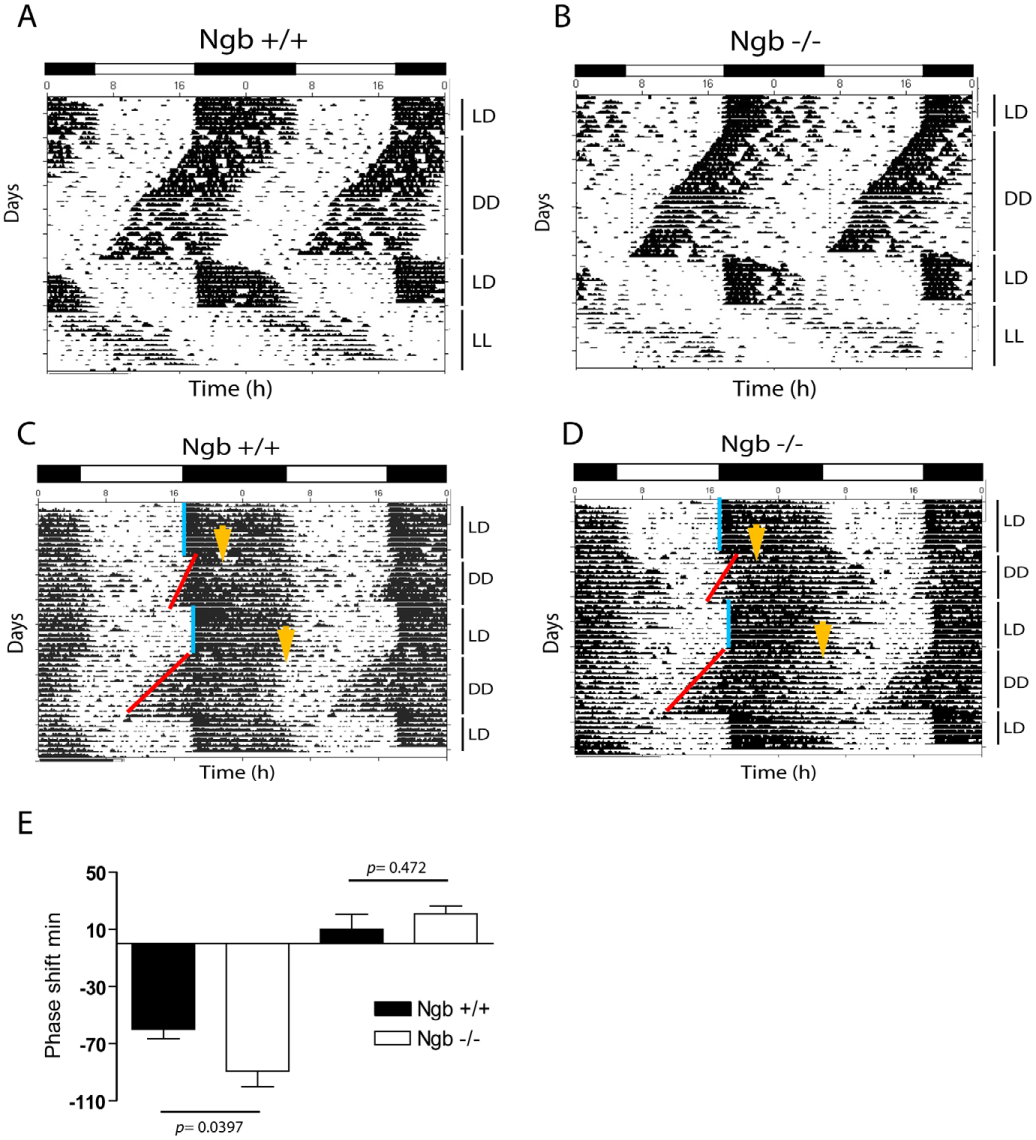
Running wheel activity in Ngb deficient mice. **A**. Double plot actogram representing running wheel activity of Ngb wild type (**A**) and Ngb deficient mice (**B**) during a period of 12:12 h LD cycle followed by constant darkness (DD), re-entrainment to a new LD cycle followed by a period of constant light (LL). Both groups behave similarly and have a TAU not significantly different between the two genotypes. **C** and **D**. Light stimulation at ZT16 and ZT22 result in phase delays and phase advance, respectively which is illustrated in wild type animals (**C**) and in Ngb deficient mice (**D**). Ngb deficient mice display a significantly larger phase delay at ZT16 as shown in E. Yellow arrows indicates the time of the light pulse. Error bars = S.E.M., Mann Whitney-U test.

**Figure 3:**
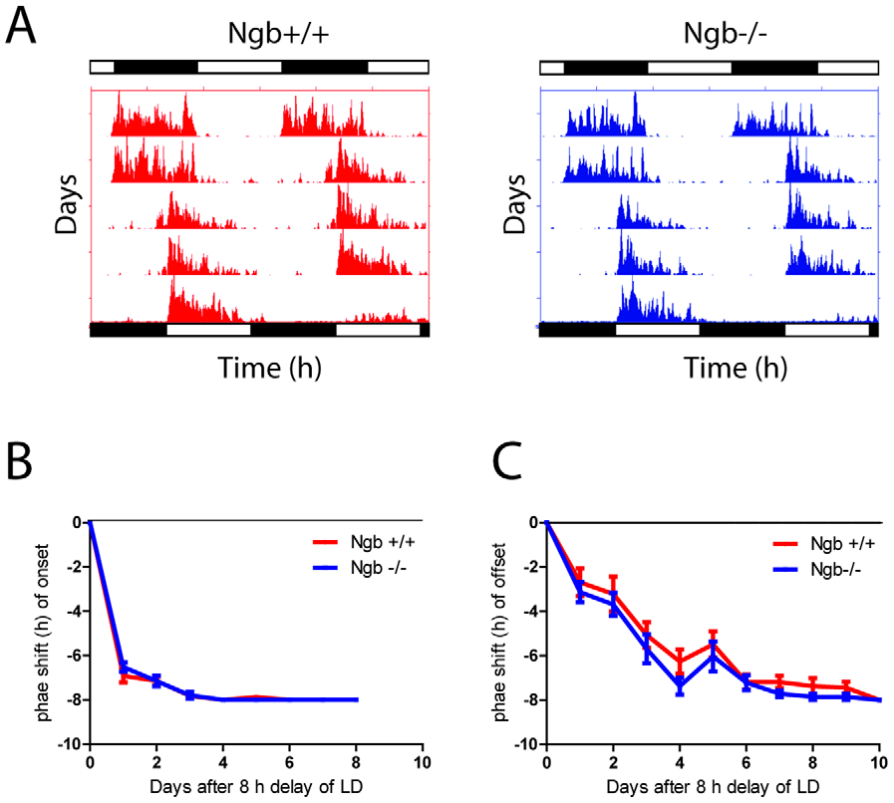
Re-entrainment after eight h phase shift of the LD cycle (jetlag) in Ngb deficient mice. **A**. Representative actogram of Ngb wild type (red) and Ngb deficient mice (blue) entrained to a 12:12 h LD cycled followed by a eight h shift (delay) of the LD cycle. Bars in top of each actogram represent the LD cycle before the shift, the bars below the LD cycle after the shift. **B**. Quantitative analysis of the eight h phase delay of the LD cycle using the onset as phase marker (n = 7 of each genotype). Note that both groups re-entrain within three cycles. **C**. Quantitative analysis of the eight h phase delay of the LD cycle using the offset as phase marker (n = 7 of each genotype). Note both groups re-entrain within seven cycles.

Ngb^−/−^ mice had similar entrainment to the LD-phase as wt mice (Fig. 2A–B) when placed in a 24 h LD cycle and, similarly to the wild type animals, they were mostly active during the dark phase (Table 1). The rhythmic behaviour of Ngb^−/−^ mice continued with an onset and τ consistent with the wild type mice when placed in constant darkness (23.83±0.06 h vs. 23.73±0.07 h) (Table 1 and Fig. 2A–B). Under constant light, the τ of both wild type and Ngb^−/−^ mice was prolonged with no significant differences between the two groups (25.06±0.11 h vs. 25.13±0.07 h) (Fig. 2A–B). There was no significant difference between the genotypes in terms of total running wheel activity both in DD and LL (Table 1). When exposed to a light pulse at ZT16, Ngb^−/−^ mice had a significantly larger phase delay of the circadian rhythm when compared to wt (89.3±11.0 min vs. 60.0±7.0 min) (Fig. 2C–E). No significant difference was observed between the genotypes after light-stimulation at ZT22 (10.0±10.4 min vs. 20.8±5.5 min) (Fig. 2C–E). Since Ngb^−/−^ mice respond to a light pulse with a larger phase shift at early subjective night, we investigated whether they re-entrain faster than wt to an 8-h phase delay of the LD cycle. Both groups used the same number of cycles to re-entrain to the new LD cycle (Fig. 3A–C).

**Table 1:** Summary of activity data.

Wheel running	Wild-type N = 8 (♂)	Ngb^−/−^ N = 8 (♂)	*p* values
τ (DD), h	23.73±0.07	23.83±0.06	ns
τ (LL), h	25.13±0.07	25.06±0.11	ns
Daytime activity	4063±758	2939±552	ns
Nighttime activity	18399±4232	16210±2339	ns
Total activity	22462±4990	19149±2891	ns
Total activity (DD)	26189±4743	23246±2525	ns
Total activity (LL)	5704±174	4251±849	ns

### Light-induced Per1 gene expression at early night

**Figure 4:**
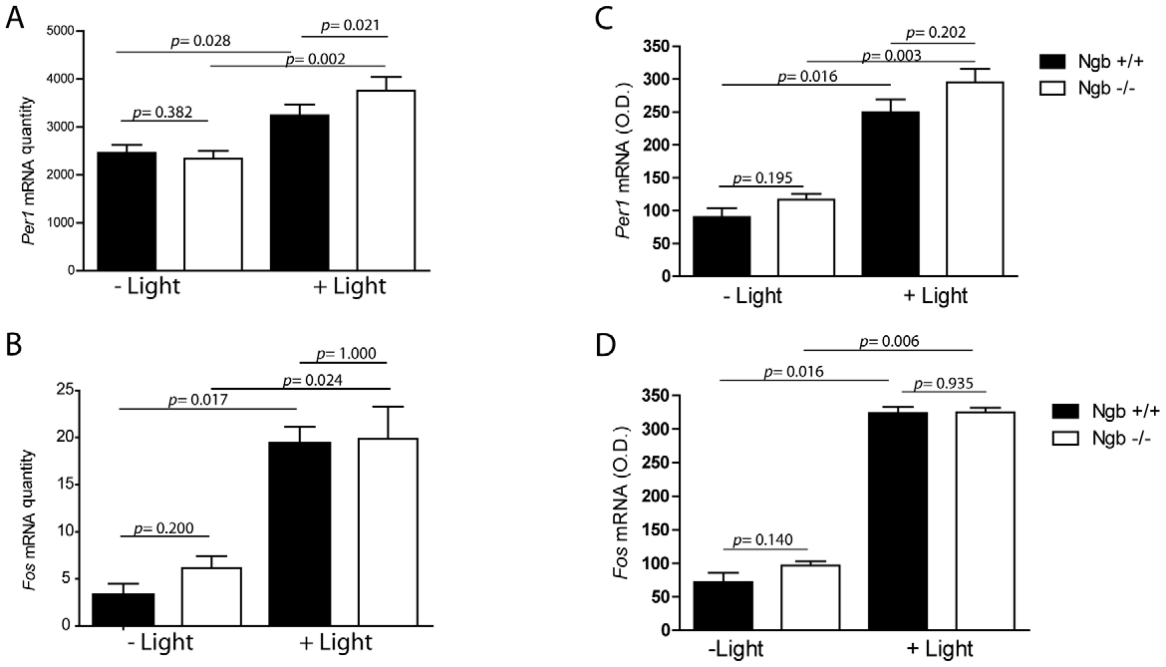
Light induced expression of *Per1* (A and C) and *cFos* (B and D) mRNA in Ngb deficient mice during early night. Left panels (A–B) show the results of quantitative analysis using RT-PCR on SCN tissue ((mRNA/ß2MG mRNA (arbitrary units), see material and methods) and the results in the right panel (C and D) are obtained by semi-quantitative in situ hybridization (optical density, see material and methods). **A**–**B**. A 30 min light pulse induced *Per1* expression in Ngb deficient mice compared to wild type controls which is significant determined by RT-PCR on SCN tissue. Light stimulation significantly induces *cFos* expression in both genotypes but no difference was found in between the two genotypes (**B** and **D**). Error bars = S.E.M., Mann Whitney-U test.

Light induced resetting of the clock involves an induction of the core clock gene *Per1* [19], [20]. We therefore investigated the light-induced expression of *Per1* and *Fos* (another light-responsive gene in the SCN [21]) genes in wt and Ngb^−/−^ mice at early subjective night where the Ngb deficient mice demonstrated a larger phase delay compared to wild type mice. Both genotypes responded to light stimulation with a significant increase in *Per1* and *Fos* mRNA expression in the SCN using two independent methods (real time RT-PCR and quantitative ISH). In relation to wt, light induced a slight but significantly higher expression of *Per1* in Ngb^−/−^ mice as determined by real-time RT-PCR (Fig. 4A). The induction of *Fos* did not differ between the genotypes (Fig. 4B). A similar trend was observed by quantitative ISH although the induction of *Per1* in Ngb^−/−^ mice did not reach statistical significance (Fig. 4C–D).

### FOS and PER1 expression in Ngb containing neurons after light stimulation at night

**Figure 5:**
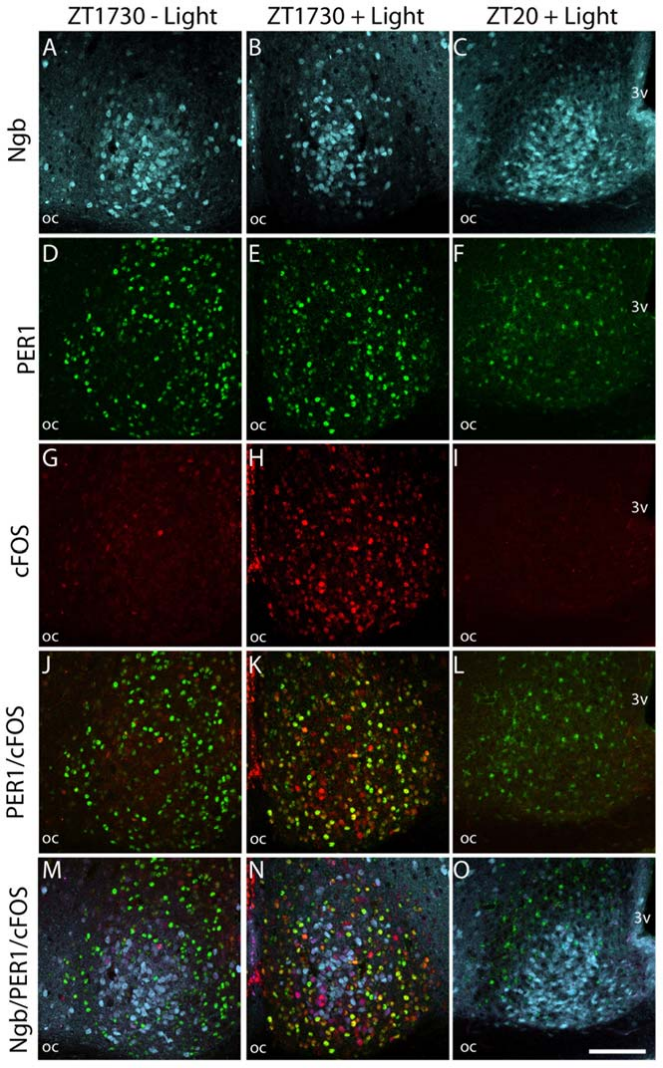
Light induced cFOS and PER1 in Ngb expressing neurons of the mouse SCN. The first column shows sections of mid SCN from a mouse euthanized at ZT 1730 without light stimulation. Ngb-IR (light blue) **A**, PER1 (green) **D** and cFOS (red) **G** are shown. Expression of Ngb-IR and PER1, but not cFOS-IR can been observed. Merged images **J–M** shows PER1/cFOS and PER1/cFOS/Ngb-IR, respectively. There was no co-expression between PER1 and Ngb. Similarly, in the mid column SCN from a mouse euthanized 90 min after light stimuli is shown. Note strong expression of cFOS (**H**) and a high degree of co-localization (yellow) with PER1-IR (**K**). No co-expression could be observed between Ngb and PER1-IR, but a subpopulation of Ngb-IR cells in the core was found to be cFOS positive (**N**). In the last column SCN from a mouse euthanized at ZT 20 after having received a 30 min light stimulus at ZT 16 is shown. Note cFOS-IR has disappeared (**I**) and substantially less PER1-IR (**L**) is expressed in both the nuclei and cytoplasm. No co-expression between Ngb-IR and PER1-IR could be seen (**O**). Oc; optic chiasm; 3 V, 3^rd^ ventricle. Scale bar 100 µm.

To investigate whether light stimulation targeted the Ngb-IR cells directly we examined the expression of PER1 in Ngb-expressing cells after 30 min light stimulation at early night. *Per1* gene expression is markedly induced after light stimulation [19] and occurs in the SCN within 30 min after light stimulation peaking after 60–120 min [19]. The exact time point for PER1 protein is less well defined in the literature. We examined animals 90 min and 240 min after light stimulation and found that in control animals PER1 is expressed in a large number of neurons not containing Ngb (Fig. 5A, D, M). Only a single FOS positive cell was found in control animals without light stimulation (Fig. 5G). FOS immunoreactivity was strongly induced in the SCN 90 min after the initiation of a light pulse (Fig. 5H) and it was co-localized with PER1-ir (Fig. 5K). Ngb neurons containing FOS-IR after a light pulse did not harbour PER1-IR (Fig. 5N). Four hours after the light pulse, FOS was no longer visible in the SCN (Fig. 5I) and PER1-IR was markedly reduced and appeared to be located primarily in the cytoplasm compared to the nuclear distribution observed at ZT1730 (Fig. 5L). No PER1 immunoreactivity could be detected in Ngb neurons in control animals (Fig. 5M) or in Ngb neurons from animals euthanized at ZT12, the time point of maximal PER1 expression in the shell region during a normal LD cycle (Fig. S1).

### Ngb-expressing neurons in the SCN are innervated from the eye and the intergeniculate leaflet (IGL)

**Figure 6:**
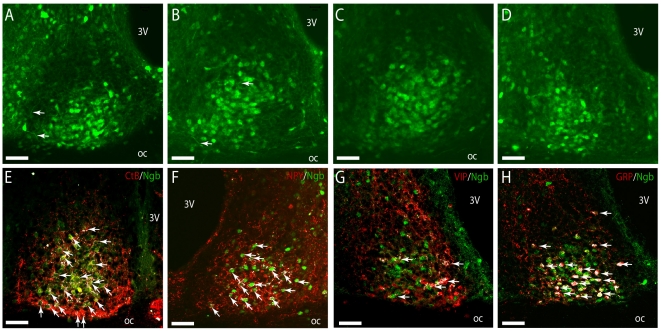
Innervation and co-expression of Ngb in the mouse SCN. In **A–D** Ngb-IR (green) can be seen in both the neuronal cell body and processes throughout the mid and ventral part of the SCN. Highest expression of Ngb-IR was observed in the mid/core part of SCN from rostral (**A**), mid (**B–C**) and in the ventral-lateral part in the caudal SCN (**D**). In (**E**) visual input from the RHT is depicted with cholera toxin subunit B (Ctb) (red). A high degree of innervation of Ngb-IR cells (green) was observed in the ventral and mid part of the SCN shown with white colour (arrows). Likewise Ngb-IR cells were also innervated by NPY-IR fibres (red) originating from the GHT (arrows) (**F**). In colchicine treated mice no co-expression of Ngb-IR and VIP-IR could be seen (**G**). Most GRP-IR cells were seen to co-express Ngb-IR (arrows) in colchicine treated mice (**H**). Oc; optic chiasm; 3 V, 3^rd^ ventricle. Scale bars 50 µm.

Ngb-immunoreactive (Ngb-IR) neurons were clustered in the ventral SCN extending from the rostral throughout the mid and caudal parts of the SCN (Fig. 6A–D). Ngb-IR was strongly expressed in the cytoplasm whereas weaker staining was found in the processes (arrows in Fig. 6 A–B). Using CtB-tracing and immunostaining retinal projections were found to innervate Ngb-expressing neurons in the lateral and ventral part of the rostral and mid SCN (Fig. 6E). Ngb expressing cells also received input from the IGL as visualized by NPY-immunoreactive nerve fibres in the ventrolateral part of the SCN (Fig. 6F). An overlap of the retinal and IGL projections was observed. The ventro-lateral SCN contains neurons expressing VIP and GRP. We found approximately half of the Ngb neurons located in the ventral part of the rostral and mid SCN co-expressing GRP immunoreactivity (Fig. 6H). As in the rat SCN [15], no Ngb-positive neurons co-storing VIP (Fig. 6G) or vasopressin (AVP) were identified (Fig. S2).

## Discussion

Previous studies have suggested that Ngb is an oxygen binding protein [13], [16] acting as an oxygen reservoir for neurons with a high metabolic demand [13] and that it is involved in protection against neuronal death (for review [18]). However, the precise functional role of Ngb remains to be determined. Anatomical studies in rodents have shown Ngb expression to be located in distinct neuronal populations in the central nervous system including the SCN [14], [15], [22]. The presence of Ngb-immunoreactivity in the SCN prompted us to examine the outcome of Ngb deficiency on the regulation of circadian behaviour by studying locomotor activity in Ngb deficient mice under various conditions of light and darkness. We found that Ngb-deficient mice could entrain to the LD cycle as their littermate controls and furthermore, they had a clock-controlled free-running rhythm with a τ similar to wt mice. These observations indicate that Ngb deficiency has little if any impact on core clock functions. Rhythmic control of behaviour and physiology emanates from clock neurons located in the shell-region many of which express AVP and clock genes [23]. Ngb neurons were observed to belong to a subpopulation of neurons, which did not exhibit rhythmic PER1 expression. Ngb-deficient mice manifested normal entrainment when placed in a 12:12 LD cycle, but their response to a phase delaying light pulse at early night was significantly larger compared to wt mice. However, Ngb knockout mice re-entrained using the same number of cycles after eight hour delay in the external LD cycle. Light induced phase shift is a property of the clock which prevents τ deviation from the astronomical day of 24 h [24]. In the Ngb deficient mice τ was not significantly different from the wt mice and the daily phase shift needed to stay entrained with the LD cycle was approximately 0.2 h. Re-entrainment after 8 h delay was, however, similar in the genotypes both when onset or offset of activity were used as markers for the phase shift. Thus, both groups were phase shifted and had normal masking behaviour as evidenced by a gradually prolonged running wheel activity in the dark phase during the cycles following the eight h shift of the external LD cycle. Interestingly, mice lacking calbindin, a protein involved in calcium signalling [25] were shown to display a similar behaviour exhibiting a significantly increased phase delay after light stimulation at early night with intact light entrainment [25]. However, another mouse model in which calbindin was genetically ablated showed a different behavioural response to light indicating that the role of calbindin is complex [26].

Previous studies have shown that *Per1* is a light-responsive clock gene and its expression level correlates with the amplitude of light-induced phase shift [20]. The larger phase delay observed in Ngb deficient mice was accompanied by an increased *Per1* expression which is in line with these observations. We found that whereas light induction of cFOS occurs in Ngb expression neurons, light induced PER1 expression occurs in non-Ngb containing neurons, some of which co-express cFOS. We thus speculate that the role of Ngb in light induced resetting must be upstream from the light induced PER1 neurons. In mice, many of the Ngb expressing neurons are innervated from the retina, co-storing GRP and express FOS upon light stimulation. Application of GRP to SCN brain culture or injection of GRP into the SCN has demonstrated a light like effect of GRP on SCN phase shifts and *Per* gene expression. Furthermore, a role the GRP receptor in light induced resetting of the clock has been established [10], [11], [27]–[31]. Thus, the occurrence of Ngb in GRP containing neurons could imply that Ngb is involved in GRP mediated light signalling via GRP receptors located on neurons in the dorso-medial SCN [10], [11], [31]. The phase shifting effects of GRP are dose-dependent [27] and lack of Ngb could alter the light sensitivity of GRP neurons leading to increased release of neurotransmitters from GRP neurons.

The altered light induced phase shift in Ngb deficient mice could also be mediated via the GHT and NPY signals since Ngb neurons in the mouse SCN, as observed in rats [15], were innervated by NPY containing nerve fibres most likely derived from the IGL. This projection mediates both indirect photic and non-photic information to the SCN [5]. NPY has in addition to its capacity to induce phase shifts during the daytime, as a result of non-photic stimulation, also blocking effects on light induced phase shifts and *Per* gene expression [32](review in [33] via the NPY Y5 receptor [34]. Furthermore, NPY blocks GRP induced phase delays at early night [35]. Ngb is found downstream for NPY signalling and at least some of the Ngb neurons co-storing GRP seem to be innervated by NPY. Lack of Ngb may therefore decrease the inhibitory signals mediated from NPY containing nerve fibres.

### Conclusion

In conclusion the present study demonstrates for the first time that Ngb, a recent member of the vertebrate heme-globin family, is highly expressed in mouse SCN neurons, some of which are light -responsive and share phenotype with light-responsive GRP neurons. Genetic elimination of Ngb does not affect core clock function but results in higher light-responsiveness as indicated by increased *Per1* gene expression in the SCN and in a larger light induced phase delay at early night. Future studies should be directed at investigating the possible role of Ngb in regulation of photoentrainment via mechanisms involving GRP and NPY signalling.

## Materials and Methods

### Generation of Ngb deficient mice

Development of the *Ngb* knockout mouse model was performed by genOway (Lyon, France) under the project number genOway/SST/HSA1-*Ngb*/260307 as described in [36]. In brief, homology regions covering 5.9 kb upstream of Ngb exon 2 and 2 kb downstream of exon 3 were subcloned from a miniBAC clone #1641C8 from 129Sv/Pas mouse genomic BAC library. FRT-flanked Neo resistance positive selection cassette was inserted downstream of exon 3 and two loxP sites were introduced upstream of exon 2 and downstream of exon 3, respectively (Fig. 1A). The targeting construct was introduced into the mouse genome by homologous recombination in 129Sv/Pas embryonic stem (ES) cells and recombinant clones were isolated by resistance to gancyclovir. Germline chimeras were obtained by injection of recombinant ES cells into C57BL/6 blastocysts. Chimeric mice were crossed with Flp recombinase-expressing mice to remove the Neo resistance cassette and obtain F1 founder mice (Fig. 1A). F1 founder mice were crossed with mice expressing Cre recombinase under the cytomegalovirus promoter [37], which resulted in the genomic deletion of Ngb exons 2 and 3 in all tissues studied (Fig. 1 B–C). The heterozygous Ngb-deficient founder mice (N2 generation of C57Bl/6J backcross) were further backcrossed with wild-type C57Bl/6J mice for 6 generations and the offspring were monitored for the absence of *Cre*-allele by PCR from tail biopsies (please see ref. [36] for primer sequences). Thus, the Ngb-deficient mouse strain used for this study was negative for the Cre allele and it was backcrossed to the C57Bl/6J genomic background for 8 generations (see figure 1 and figure legend).

### Behavioural studies of circadian rhythm

#### Activity rhythms

Seven male Ngb deficient (Ngb^−/−^) and seven male wild type littermate (wt) mice of 9–12 weeks of age when initiating the experiment were housed individually in cages equipped with a running wheel in ventilated, light-tight chambers with controlled white lighting. Wheel running activity was monitored by an on-line PC connected via a magnetic switch to the Minimitter Running Wheel activity system (consisting of QA-4 activity input modules, DP-24 dataports and Vital View data acquisition system, MiniMitter Company, Inc. Sunriver, OR, USA vers. 4.1) [38]. Wheel revolutions were collected continuously in 10 min bins. Animals were entrained to a 12:12 LD cycle (lights on at 7:00 a.m. designated Zeitgeber time (ZT) = 0, off at 7.00 p.m. = ZT12) at 300 lux for at least 14 d prior to the initiation of experiments. White lightning was delivered from fluorescent tubes placed on top of each cage. The light intensity was measured using an Advantest Optical Power meter TQ8210 (MetricTest, Hayward, CA), having an intensity at the cage of 300 lux (correspond to 115.0 µW/cm^2^ measured at 514 nm).

#### Endogenous Period TAU (τ)

Free-running period (τ) was assessed during days 4–18 in constant darkness (DD) or in constant light (LL) after re-entrainment to an LD cycle. TAU was calculated using χ^2^ periodogram in ClockLab (ActiMetric Software, Coulbourn Instruments, Wilmette, IL, USA).

### Light induced phase shift using Aschoff type II regime

Light induced phase shift of the circadian rhythm was determined using the Aschoff type II regime as described previously [39]. All animals were light stimulated for 30 min at 300 lux in their home-cages in separate experiments at ZT16 and ZT22, respectively, where after the lights were turned off for the next 10–14 d followed by 14 d of re-entrainment in LD before the next light pulse experiment. The light induced phase shift was determined as described previously using the difference in phase from regression lines drawn through the activity onset of the entrained (LD) onset immediately before the day of stimulation and the onset from two-three d after light stimulation of the free running activity onsets (DD) (to avoid any mislead due to transients) [39].

### 8 h phase delays (jetlag) evaluated using running wheel activity

Since Ngb mice show altered responsiveness to light pulse at early subjective night (see below), we investigated whether the changed sensitivity to light influenced the time of re-entrainment during an 8 h phase delay of the external LD cycle. Re-entrainment was defined as the first day of consecutive days in which the onset occurred within 30 min in phase with the new LD cycle.

### Light induced gene expression at early night

To elucidate the mechanism involved in the altered light induced phase shift found in Ngb deficient mice at early subjective night, we used two different methods to quantify light induced gene expression in the SCN. The wt mice used in this and the following part of the study were not littermates. We first examined a group of Ngb^−/−^ and wt mice (n = 11 and n = 8, respectively), which received a 90 min light pulse at ZT16 and a control group of Ngb^−/−^ and wt mice (n = 8 and n = 7, respectively) euthanized in dim red light; all animals were decapitated at ZT17.30. These animals had their brains removed and frozen on dry ice where after the SCN's were dissected and RNA extracted as described previously [40]. *Per1* and *Fos* mRNA was quantified by real time RT-PCR using the TaqMan gene expression assays: Mm00501813_m1 (*Per1*) and Mm00487425_M1 (*Fos*) (Applied Biosystems, Carlsbad, USA) with β2-microglobulin mRNA as internal control and standard curves by serial dilutions of cDNA as described previously [40]. Another group of light stimulated male Ngb^−/−^ and wt mice (n = 7 and n = 5, respectively) and a control group of Ngb^−/−^ and wt mice (n = 8 and n = 7, respectively) were euthanized in dim red light and decapitated at ZT17. 30 mice had their brains removed, frozen and cut in coronal sections through the SCN. These sections were processed for *in situ* hybridization histochemistry (ISH) and light induced gene expression for *Fos* and *Per1* mRNA were determined as described previously [38].

### Characterization of Ngb expression neurons in the mouse SCN

#### Animals

To investigate the anatomical localization, retinal innervation and responsiveness to light stimulation a series of anatomical studies were performed in wild type C57Bl/6J mice (in house breeding). All animals were housed under a standard 12 h light: 12 h dark (LD12:12) photoperiod (lights-on at 06:00; ZT0), anaesthetized using subcutaneous administered Hypnorm/Midazolam as described below and perfusion fixed using Stefanini fixative [15]. The brains were removed and post-fixed in the same fixative overnight, cryoprotected in 30% sucrose-PBS for five days, frozen and sectioned in 40 µm thick coronal slices in replicas of three. Ten wild type male mice were used to characterize Ngb expressing neurons in the mouse SCN and were fixed during the subjective day (ZT4-ZT12). Of these five were pre-treated with intracerebroventricular (icv) injection of the mitosis inhibitor (and axoplasmic transport-blocker) colchicine under Hypnorm/Midazolam anaesthesia as described below followed by postoperative antibiotic and pain treatment (Baytril® vet, 5 mg/kg and Rimadyl 5 mg/kg). Briefly, colchicine injections 3 µl (dissolved in 0.9% NaCl to a final concentration of 10 mg/ml) were slowly infused into the lateral ventricle using a Hamilton syringe with a 26G needle attached. Injection coordinates were: AP - 0.2 mm from bregma, L 1.2 mm from midline, and V 2.3 mm deep to the surface of the brain, according to Paxinos and Franklin (2001). The syringe was left in the brain for three minutes after injection to prevent back-flow of the colchicine. Thirty-six hours later animals were anesthetized and perfused with Stefanini fixative, cryoprotected and stored at −80°C until processed for immunohistochemistry. Three male mice were used to examine retinal innervation of Ngb expressing neurons of the SCN. These animals were anesthetized by subcutaneous injections of a mixture of fentanyl (0.20 mg/kg body weight (BW)), fluanisone (6.25 mg/kg BW) and midazolam (3.13 mg/kg BW) where after each animal received bilateral intravitreal injections of Choleratoxin Subunit B (CtB) conjugated to Alexa594 (3 µL of 1 µg/µL of CtB in PBS; Molecular Probes, Eugene, OR, USA). A seven day transport time was provided before fixating the animals. Three male mice were perfusion fixed at ZT17.30 after receiving a light pulse (300 lux of white light) at ZT16 to study light responsive gene expression (*FOS* and *PER1*) in Ngb containing SCN neurons. Control mice (n = 2) not receiving a light pulse were perfusion fixed simultaneously. Another three male mice were perfusion fixed at ZT20 after receiving a light pulse (300 lux of white light) at ZT16 to study light responsive gene expression (*FOS* and *PER1*) in Ngb containing SCN neurons after 4 h. Control mice (n = 2) not receiving a light pulse were perfusion fixed simultaneously. Animal care and all experimental procedures were conducted in accordance to the principles of Laboratory Animal Care (Law on Animal Experiments in Denmark, publication 1306, November 23, 2007) and Dyreforsoegstilsynet, Ministry of Justice, Denmark, who issued the licence number 2008/561-1445 to Jan Fahrenkrug and thereby approving the study.

### Immunohistochemistry

The primary antibodies used in the present study are presented in Table 2. For double immunostaining, Ngb was detected by using a rabbit polyclonal antibody raised against purified recombinant mouse Ngb [15]. The Ngb antibody was visualized by a biotinylated donkey anti-rabbit (Fab)_2_ (code no:711-066-152 Jackson Immunoresearch Laboratories, Baltimore, PA, USA, diluted 1∶800) in combination with Avidin-Biotin-peroxidase Complex (ABC) (VWR international, Roedovre Denmark), followed by biotinylated tyramide (Tyramide System Amplification, PerkinElmer Waltham, MA, USA) and streptavidin-488 (code no: 016-488-084 Jackson Immunoresearch Laboratories, Baltimore, PA, USA, diluted 1∶500) [15]. Other primary antibodies used in combination with the Ngb antibodies were visualized with either donkey anti goat or anti rabbit Alexa-594 (code no: A-11058 or A-21209 Molecular Probes, USA, diluted 1∶800), donkey anti rabbit Alexa-647 or donkey anti guinea pig Dylight-594 (code no: 711-606-152, 706-506-148 Jackson Immunoresearch Laboratories, Baltimore, PA, USA, diluted 1∶500). As a control the primary antibodies were omitted, which eliminated all staining from the corresponding secondary antibodies. The Ngb antibody demonstrates no specific staining when applied on brain sections from Ngb deficient mice (Fig. 1B, in replicas of three) or subjected to Western Blotting containing brain tissue from Ngb deficient mice (see below).

**Table 2:** Antibodies.

Molecular marker	Antibody	Working dilution	Source
Arginine vasopressin (AVP)	Guinea pig, polyclonal Immungen: H-Cys-Tyr-Phe-Gln-Asn-Pro-Arg-Gly-NIH_2_	1∶2000	Peninsula Laboratories, CA, USA, code no: T-5048
FOS	Goat, polyclonal Immunogen: N-terminus of cFOS of human origin	1∶100	Santa Cruz Biotechnology, CA, USA, code no: SC-52-G
Gastrin releasing peptide (GRP)	Rabbit, polyclonal. Immunogen: Synthetic peptide corresponding aa 1–27 of GRP	1∶500	A gift from Professor J. J. Holst, The Panum Institute, Copenhagen University. Lot 1267-3 [43].
Neuroglobin	Rabbit, polyclonal Immunogen: purified recombinant mouse Neuroglobin	1∶100.000; 1∶300.000	Code: 4836/5. In house
Neuropeptide Y (NPY)	Goat, polyclonal Immunogen: rat NPY1-36	1∶1000	A gift from Phillip Just Larsen, The Panum Institute, Copenhagen University,
PERIODE 1 (PER1)	Rabbit, polyclonal. Immunogen: The N-terminal part of mouse Per1 (404 amino acids)	1∶8000	In house. Code S298 [44]
Vasoactive intestinal peptide (VIP)	Rabbit, polyclonal Immunogen VIP1-28	1∶1000	In house code 291E-5 [45]
Cholera toxin subunit B (CtB)	Goat, polyclonal #703, (Lot #7032E)	1∶1000	List Biological Laboratories, Campbell, USA.

To evaluate possible contacts or co-localization between Ngb immunoreactive nerve fibres and cell bodies and input from the retina (CtB positive retinal projections) or indirect light input via the GHT (NeuropeptideY (NPY) immunoreactive nerve fibres)) photomicrographs were obtained using an Olympus IX70 confocal microscope equipped with Fluorview (vers. 2.1.39, Olympus, Denmark) or a Zeiss LSM 780 on Axio Observer (Zeiss, Denmark) and appropriate filter settings for detecting Alexa488, Alexa568 and Alexa647 fluorophores and possible contacts were estimated using the co-localization plugin in ImageJ software (vers. 1.42q, NIH, USA) at the rostral, mid and caudal levels of the SCN. Program default values (Display value = 255; Channel threshold 50%) were used when making the estimates.

### Extraction and immunoprecipitation of Neuroglobin

Wild type and Ngb deficient mice were euthanized by decapitation and the brains rapidly removed and placed on ice. The brain was cut through the midbrain below the dorsal 3^rd^ ventricle. Cortex and the cerebellum were removed to generate hypothalamus-enriched tissue, which exhibit high expression of Ngb [41]. The hypothalamic enriched tissue was frozen on dry-ice and stored at −80°C until extraction of Ngb. Following addition of 1 mL ice-cold immunoprecipitation buffer (IP buffer) containing: 50 mM Tris pH 7.4, 150 mM NaCl, 1 mM EDTA, 1% NP-40 supplemented with 1% Halt Phosphatase Inhibitor Cocktail (Pierce, Rockford, IL, USA) and protease inhibitors (Roche Mini EDTA-free Complete® tablet), frozen hypothalamus-enriched tissue was homogenized with the aid of 10 strokes of a pellet pestle and a sterile scalpel. After 30 min of lyses on ice, extracts were cleared for insoluble material at 15,000× *g* for 10 min at 4°C and transferred to clean tubes. To reduce background from endogenous immunoglobulin, the extracts were pre-cleared with 50 µl 50% protein G-Sepharose slurry (Amersham, GE Healthcare, USA) for 1 h at 4°C. Each sample consisting of 900 µl hypothalamus-enriched proteins from either wild type or Ngb^−/−^ mice was divided into two clean tubes. Ngb was immunoprecipitated by adding 4 µl rabbit anti-Ngb antiserum (In house generated Code#RbNGB 4836/5) characterized in [42] the tube and incubation overnight at 4°C. Antibody-Ngb complex was captured by incubation with 60 µl 50% protein A-Sepharose slurry (Amersham, GE Healthcare, USA) for 1 h at 4°C. Beads were washed three times with 1 ml IP buffer and stored as wet pellets at −80°C until Western blotting.

### Western blotting

All reagents and equipment used for electrophoresis and transfer of proteins were used according to manufacturer's instructions regarding the NuPAGE® system (Invitrogen). Frozen beads were briefly thawed on ice and proteins were eluted in 60 µl 2X SDS sample buffer (100 mM Tris (pH 6,8), 8% SDS, 24% glycerol, 80 mM HCl and 0,025% Coomasie brilliant blue) freshly supplemented with 1X NuPAGE Reducing agent (Invitrogen, Carlsbad, CA, USA) for 10 min at 85°C. Samples were centrifuged at room temperature for 1 min at 10,000× *g* and eluted proteins were transferred to clean tubes. Equal amounts (20 µl) of each sample were immunoblotted for immunoprecipitated Ngb as described below. Recombinant Ngb [17] was used as a positive control. Immunoblotting was done overnight at 4°C with previously characterized rabbit Ngb antiserum (diluted 1∶5000; (Hundahl et al., 2010)). Immunoreactivity was detected with a conformation-specific mouse anti-rabbit IgG (diluted 1∶2000, cat.no: 3678; Cell Signaling Technology, Beverly, MA, USA) and an anti-mouse IgG horseradish peroxidase-conjugated antibody (diluted 1∶2500; Dako, Glostrup, Denmark). Protein bands were visualized with enhanced chemiluminescence according to manufacturer's protocol (Western Lightning®Plus-ECL, PerkinElmer, Waltham, MA, USA). Images of developed films were adjusted for contrast and brightness in Photoshop (Adobe). The western blotting experiment was conducted in replicas of three.

### Statistics

Statistical analysis was performed with GraphPad Prism v 4.0. Non-parametric statistics (Mann Whitney U test) was used for comparisons between two groups since the criteria for using parametric statistic (data followed a normal distribution) was not fulfilled in this material. *p*<0.05 was considered statistically significant.

## Supporting Information

Figure S1 **PER1 and Ngb expression in the mouse SCN.** In **A** strong Neuroglobin (Ngb) green and Period1 (PER1) red immunoreactivity (IR) is seen in the mid part of the suprachismatic nucleus (SCN) from a mouse euthanized at ZT12, the time point at which the PER-IR is highest. The area within the square is magnified in **B** showing no co-localization between Ngb-IR and PER1-IR. In the caudal part of the SCN (**C**) Ngb-IR and PER1-IR was also clearly separated. OC (Optic chiasm), 3 V (3ed ventricle). Scale bar 50 µm.
(TIF)

Figure S2 **Ngb expressing neurons do not express arginine-vasopressin-IR (AVP).** **A**–**B** shows Ngb-IR in green and arginine-vasopressin-IR (AVP) in red in the rostral and mid SCN, respectively. Ngb-IR and AVP-IR was clearly separated in two compartments of the SCN. OC (Optic chiasm), 3 V (3ed ventricle). Scale bar 50 µm.
(TIF)
