# Supplementary figures and images for: Circadian Behaviour in Neuroglobin Deficient Mice
Source: PLoS One. 2012 Apr 5;7(4):e34462. doi: 10.1371/journal.pone.0034462 (PMC3320642; doi:10.1371/journal.pone.0034462)

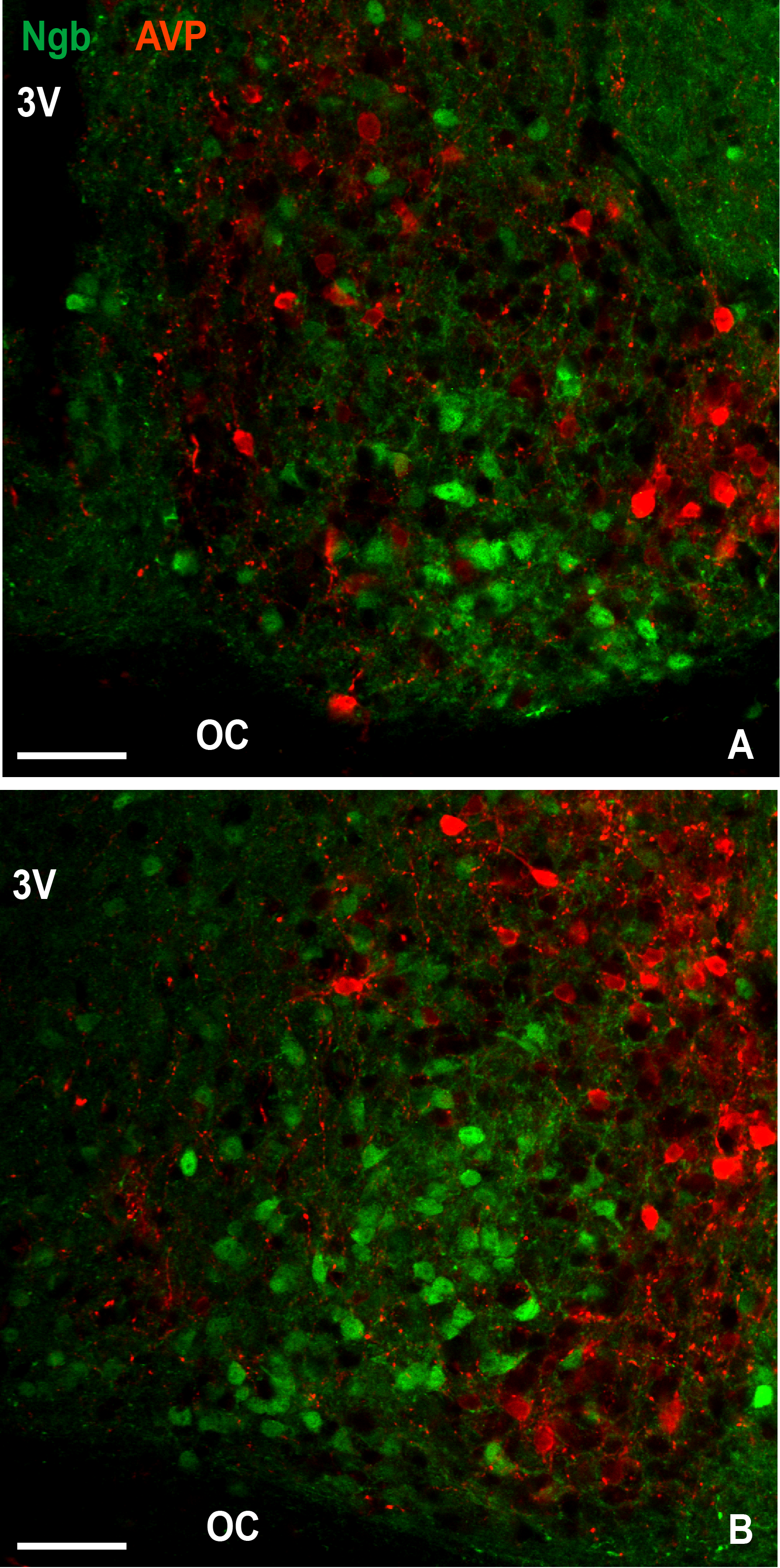

Supplement: Figure S2 — Ngb expressing neurons do not express arginine-vasopressin-IR (AVP). A–B shows Ngb-IR in green and arginine-vasopressin-IR (AVP) in red in the rostral and mid SCN, respectively. Ngb-IR and AVP-IR was clearly separated in two compartments of the SCN. OC (Optic chiasm), 3 V (3ed ventricle). Scale bar 50 µm. (TIF) [file pone.0034462.s002.tif]
